# Direct Contra Naïve-Indirect Comparison of Clinical Failure Rates between High-Viscosity GIC and Conventional Amalgam Restorations: An Empirical Study

**DOI:** 10.1371/journal.pone.0078397

**Published:** 2013-10-28

**Authors:** Steffen Mickenautsch, Veerasamy Yengopal

**Affiliations:** Systematic Review initiative for Evidence-based Minimum Intervention in Dentistry/Department of Community Dentistry, Faculty of Health Sciences, University of the Witwatersrand - Johannesburg, South Africa; University Hospital of the Albert-Ludwigs-University Freiburg, Germany

## Abstract

**Background:**

Naïve-indirect comparisons are comparisons between competing clinical interventions’ evidence from separate (uncontrolled) trials. Direct comparisons are comparisons within randomised control trials (RCTs). The objective of this empirical study is to test the null-hypothesis that trends and performance differences inferred from naïve-indirect comparisons and from direct comparisons/RCTs regarding the failure rates of amalgam and direct high-viscosity glass-ionomer cement (HVGIC) restorations in permanent posterior teeth have similar direction and magnitude.

**Methods:**

A total of 896 citations were identified through systematic literature search. From these, ten and two uncontrolled clinical longitudinal studies for HVGIC and amalgam, respectively, were included for naïve-indirect comparison and could be matched with three out twenty RCTs. Summary effects sizes were computed as Odds ratios (OR; 95% Confidence intervals) and compared with those from RCTs. Trend directions were inferred from 95% Confidence interval overlaps and direction of point estimates; magnitudes of performance differences were inferred from the median point estimates (OR) with 25% and 75% percentile range, for both types of comparison. Mann-Whitney U test was applied to test for statistically significant differences between point estimates of both comparison types.

**Results:**

Trends and performance differences inferred from naïve-indirect comparison based on evidence from uncontrolled clinical longitudinal studies and from direct comparisons based on RCT evidence are not the same. The distributions of the point estimates differed significantly for both comparison types (Mann–Whitney U  =  25, n_indirect_  =  26; n_direct_  =  8; p  =  0.0013, two-tailed).

**Conclusion:**

The null-hypothesis was rejected. Trends and performance differences inferred from either comparison between HVGIC and amalgam restorations failure rates in permanent posterior teeth are not the same. It is recommended that clinical practice guidance regarding HVGICs should rest on direct comparisons via RCTs and not on naïve-indirect comparisons based on uncontrolled longitudinal studies in order to avoid inflation of effect estimates.

## Introduction

The term ‘high-viscosity’ or ‘high-viscous glass-ionomer cement’ (HVGIC) has emerged within the scientific dental literature: A simple search conducted in PubMed/Medline (25.09.2012) with the string of search terms: "high-viscosity glass ionomer cement" OR "high-viscous glass ionomer cement" revealed 16 citations of articles, published between 2003 – 2011, of which five articles referred to the term in their titles and all articles in their listed abstracts and related it specifically to the products Fuji IX (GC Corporation, Japan) or Ketac Molar (3M ESPE, Germany).

HVGICs appear distinct from other (low) viscosity GICs (including Cermets) in their comparative survival rate to that of conventional amalgam restorations. The results of a meta-analysis found a survival rate for HVGIC (Fuji IX; Ketac Molar) similar to that of amalgam but showed significantly lower survival rates for “low-viscosity” GICs (Chelon Silver ( =  Cermet); Chem Fil; Fuji II) than for amalgam [1].

Glass ionomer cements, such as HVGICs, adhere primarily via calcium bonds to the mineral content of the tooth structure [2]. This adherence provides an adaptive seal, and, as the material slowly leaches fluoride ions into the adjacent tooth tissue, these materials are capable of halting or slowing the progression of carious lesions [3]. Glass-ionomer cements are ideally suited to managing dental caries as they can be applied in the very early stages of caries development or in the larger cavity. Additionally, they simplify the tooth restorative procedure and enable the dentine-pulp complex to react against the caries process [4].

Amalgam has been used successfully as an universal posterior restorative material for over a century [5]. Its operative advantages of being relatively simple to place, its intrinsic strength and the longevity of the final restoration has led to amalgam’s being considered the “gold standard” against which all newer materials, such as HVGICs, are measured for outcomes; such as the effectiveness and durability of the restoration.

In line with the low-/high-viscosity distinction of conventional (chemically cured) glass-ionomer cements (GICs) based on such pure clinical grounds, definition of HVGICs according to laboratory/material characteristics such as powder/liquid ratio or compressive strength may prove to be difficult i.e.: the powder/liquid ratio for Ketac Molar and Fuji IX has been reported to be 2.9/1 [6] and 3.6/1 [6], [7], respectively but appears to be not generally higher than that reported for Chelon Silver; Chem Fil and Fuji II (3.8/1 [8]; 3.7/1 [9] and 2.7/1 [7], respectively). While the measured compressive strength of HVGIC may be above 200 Mpa [10] after 24 hours, and that of low-viscosity GIC below 200 Mpa [8], [9], a further laboratory study reported the compressive strength of Fuji IX to be 147.93 Mps (SD  =  18.02) after 24 hours [11]. Such conflicting and inconclusive *in-vitro* evidence may be attributed to heterogeneous methodologies employed in different laboratory studies and thus have to be regarded with caution. In addition, caution in extrapolating *in-vitro* results to clinical practice is warranted on the basis that *in-vitro*/laboratory evidence appears to correlate poorly with the clinical merits of dental materials [12], [13].

Against this background the distinction between low and high-viscosity conventional GICs, on a clinical rather than chemical basis, seems to empirically support justification and recommendation of HVGIC as an appropriate restorative treatment option in permanent posterior teeth [1]. However, such consideration may currently not be shared by many dental associations in developed countries and may even contravene standing recommendations. In Germany, for example, the joint statement issued in 2005 by two dental associations, i.e. Deutsche Gesellschaft für Zahnerhaltung (DGZ) and Deutsche Gesellschaft für Zahn- Mund- und Kieferheilkunde (DGZMK), states that HVGICs are due to their high fracture and wear risk not suitable for use in permanent posterior tooth restoration [14].

A detailed analysis of the DGZ/DGZMK statement (File S1) reveals that its recommendations regarding HVGIC are based on the findings of one comprehensive, non-systematic literature review by Manhart et al., 2004 [15]. Although the difficulty of comparison of clinical material characteristics from uncontrolled clinical longitudinal studies is asserted in this review, the authors, however, maintain that certain trends and performance differences between for example amalgam and glass-ionomer cement restorations may be inferred from these types of studies [15]. Consequently, the review bases its content, conclusions and recommendations on restoration survival and failure rates mainly extracted from cross-sectional and uncontrolled clinical longitudinal studies and lists these results separately for amalgam-, direct composite-, compomer-, GIC-, gold, composite and ceramic inlay/onlay restorations in posterior teeth in tables for naïve-indirect comparisons [15]. (According to commonly accepted terminology ‘naïve-indirect comparison’ is defined as ‘comparison of competing clinical interventions from data of individual arms of different studies, based on the assumption that the treatment groups are clinically homogeneous in composition’. In contrast, direct comparisons are comparisons between randomised intervention groups within RCT settings [16]).

Against this background, the aim of this empirical study is to investigate whether trends and performance differences between conventional amalgam and direct HVGIC restorations in posterior teeth can be inferred through naïve-indirect comparison of failure rates from uncontrolled longitudinal clinical studies. The null-hypothesis is tested that trends and performance differences inferred from naïve-indirect comparison based on evidence from uncontrolled longitudinal clinical studies and from direct comparisons based on RCT evidence have similar direction and magnitude.

## Materials and Methods

### Search of uncontrolled clinical longitudinal studies

PubMed/Medline was searched by both authors (SM and VY), independently, following a simple, systematic search strategy. The search terms: “atraumatic restorative treatment” was used in order to identify longitudinal studies investigating HVGIC. Longitudinal studies investigating amalgam were searched using the following string of Mesh search terms: "Dental Amalgam"[Mesh] AND "Dental Restoration, Permanent"[Mesh]. The string was constructed from the terms "Dental Restoration, Permanent"[Mesh] (yielding 10434 citations) and "Dental Amalgam"[Mesh] (yielding 1274 citations). The search period was limited to publications from 2002/01/01 to 2012/09/25.

Titles and abstracts of the resulting citations were scanned for possible inclusion in line with the following inclusion criteria:

- Prospective clinical one-arm study (uncontrolled longitudinal study investigating either direct HVGIC or conventional amalgam restorations) or quasi-one-arm study (two-arm study that did not compare HVGIC with amalgam restorations, but included either HVGIC or amalgam as one of the study arms);
- Minimum 12-month follow-up period;
- Investigated cavity type Class I or II in permanent posterior teeth (Tunnel restorations not included);
- Publication language: English;
- Study outcome: restoration failure.

Articles whose title and abstracts were in alignment with the inclusion criteria were retrieved in full copy and were reviewed by both authors of this article. Disagreements were resolved through discussion and consensus. Articles were excluded if no computable data were reported or if they did not match the characteristics of the control data.

### Selection of uncontrolled clinical longitudinal studies

**Figure 1:**
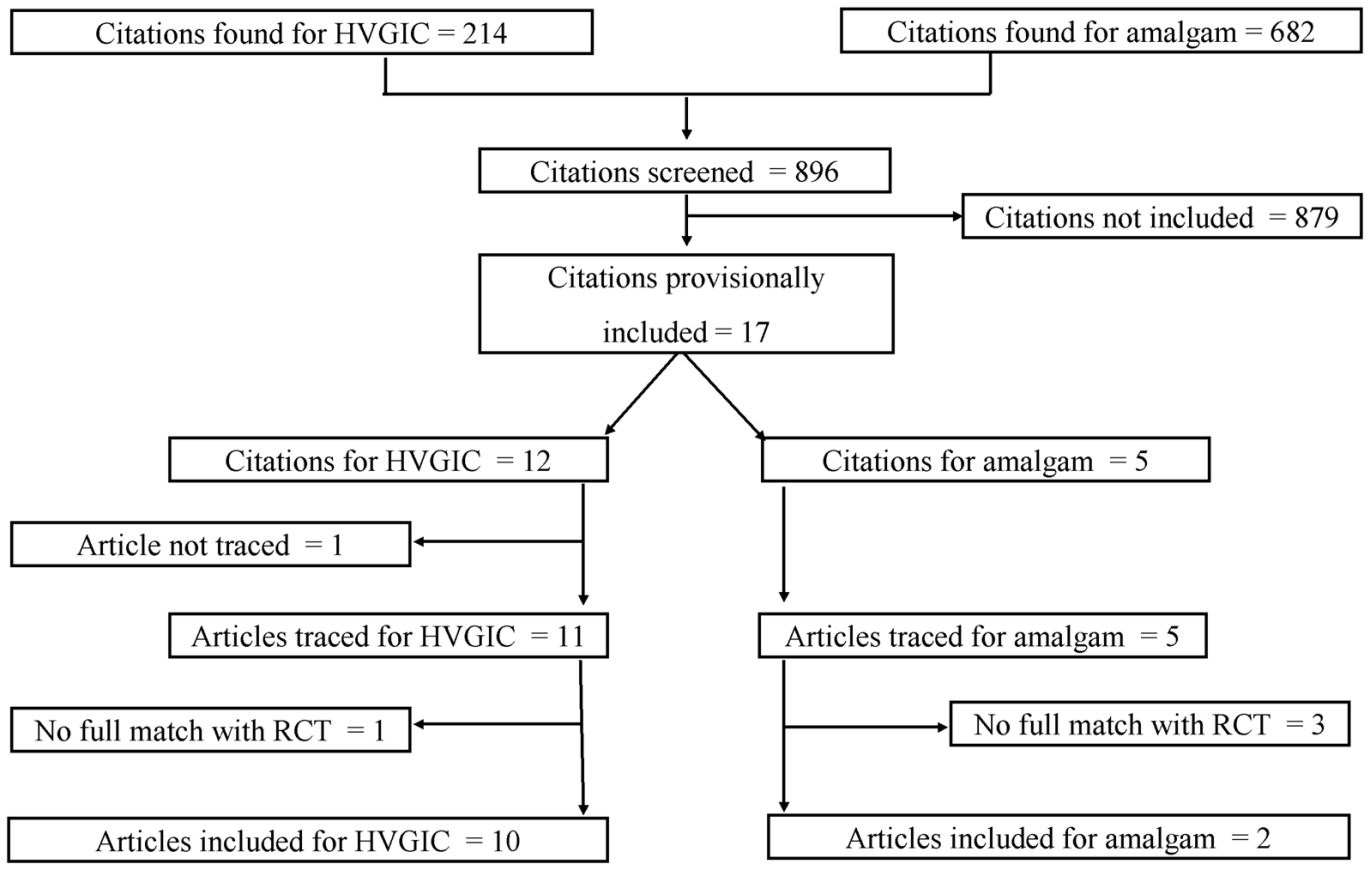
Flow diagram of uncontrolled clinical longitudinal study selection. RCT  =  Matched randomised control trials from systematic review [13]; HVGIC  =  high-viscosity glass-ionomer cement.

Figure 1 provides information on the number of uncontrolled clinical longitudinal studies identified through the search strategy. The search of PubMed/Medline generated 214 citations for HVGIC and 682 for amalgam restorations. Of these, 12 and five citations fulfilled the inclusion criteria, respectively, and were further reviewed. One article related to HVGIC [17] could not be traced in full as the journal appeared to be suspended and the article was thus excluded. In total, 11 articles related to HVGIC [18]–[28] and 5 for amalgam [29]–[33] were provisionally accepted (Table 1).

**Table 1:** Included uncontrolled clinical longitudinal studies.

High-viscosity Glass-ionomer cements (HVGIC)					
**Study**	**Reference**	**Quasi one-arm**	**Cavity class**	**Follow-up period**	**HVGIC**
Lopez et al., 2005	[18]	No	I, II	12, 24 months	Fuji IX
Gemert-Schriks et al., 2007	[19]	No	I	36 months	Ketac Molar
Zanata et al., 2011	[20]	No	I, II	12, 24, 120 months	Fuji IX
Ibiyemi et al., 2011	[21]	No	I	12, 24 months	Fuji IX
Barata et al., 2008	[22]	Yes	I, II	12 months	Ketac Molar
Ercan et al., 2009	[23]	Yes	I, II	12, 24 months	Ketac Molar
Ziraps and Honkala, 2002	[24]	Yes	I	24 months	Fuji IX/Chem Flex
Wang et al., 2004	[25]	No	I	36 months	Ketac Molar
Lo et al., 2007	[26]	No	I, II	5, 6 years	Ketac Molar
Abid et al., 2002	[27]	No	I	12, 24, 36 months	Fuji IX
Cefaly et al., 2007	[28]	Yes	I, II	12 months	Ketac Molar
Amalgam					
**Study**	**Reference**	**Quasi one-arm**	**Cavity class**	**Follow-up period**	
Sachdeo et al., 2004	[29]	Yes	II	12, 24 months	
Soncini et al., 2007	[30]	Yes	I	Mean 3.4 (SD = 1.9) years	
Bernardo et al., 2007	[31]	Yes	I, II	7 years	
Van Nieuwenhuysen et al., 2003	[32]	Yes	‘extensive’ (II)	36 months	
Kiremitci and Bolay, 2003	[33]	Yes	I	12, 24, 36 months	

SD  =  Standard deviation.

The included HVGIC and amalgam longitudinal studies were matched with each other, as well as with available RCTs [34] according to investigated cavity type and follow-up period (Table 2). No full-match was found for one HVGIC study [26] and three amalgam studies [30]–[32] due to different length of follow-up period per cavity type. These studies were thus excluded.

**Table 2:** Matched studies as per cavity class and follow-up period.

Cavity class	Follow-up period	Uncontrolled clinical longitudinal studies		RCTs
		HVGIC	Amalgam	HVGIC versus Amalgam
I	12 months	Lopez et al., 2005 [18]	Kiremitci and Bolay, 2003 [33]	Yip et al., 2002 [35]
		Zanata et al., 2011 [20]		Frencken et al., 2006 [36]
		Ibiyemi et al., 2011 [21]		
		Barata et al., 2008 [22]		
		Ercan et al., 2009 [23]		
		Abid et al., 2002 [27]		
		Cefaly et al., 2007 [28]		
	24 months	Lopez et al., 2005 [18]	Kiremitci and Bolay, 2003 [33]	Frencken et al., 2006 [36]
		Zanata et al., 2011 [20]		Rahimtoola and van Amerongen, 2002 [37]
		Ibiyemi et al., 2011 [21]		
		Barata et al., 2008 [22]		
		Ercan et al., 2009 [23]		
		Abid et al., 2002 [27]		
		Ziraps and Honkala, 2002 [24]		
	36 months	Gemert-Schriks et al., 2007 [19]	Kiremitci and Bolay, 2003 [33]	Frencken et al., 2006 [36]
		Wang et al., 2004 [25]		
		Abid et al., 2002 [27]		
	Mean 3.4 (SD = 1.9) years	(No match)	Soncini et al., 2007 [30]	(No match)
	4 years	(No match)	(No match)	Frencken et al., 2006 [36]
	5 years	Lo et al., 2007 [26]	(No match)	Frencken et al., 2006 [36]
	6 years	Lo et al., 2007 [26]	(No match)	Frencken et al., 2006 [36]
	7 years	(No match)	Bernardo et al., 2007 [31]	(No match)
	10 years	Zanata et al., 2011 [20]	(No match)	(No match)
II	12 months	Lopez et al., 2005 [18]	Sachdeo et al., 2004 [29]	Frencken et al., 2006 [36]
		Zanata et al., 2011 [20]		
		Barata et al., 2008 [22]		
		Ercan et al., 2009 [23]		
		Cefaly et al., 2007 [28]		
	24 months	Lopez et al., 2005 [18]	Sachdeo et al., 2004 [29]	Frencken et al., 2006 [36]
		Zanata et al., 2011 [20]		
		Ercan et al., 2009 [23]		
	36 months	(No match)	Van Nieuwenhuysen et al., 2003 [32]	(No match)
	5 years	Lo et al., 2007 [26]	(No match)	(No match)
	6 years	Lo et al., 2007 [26]	(No match)	(No match)
	7 years	(No match)	Bernardo et al., 2007 [31]	(No match)

SD  =  Standard deviation; HVGIC  =  High-viscosity glass-ionomer cement; RCT  =  Randomised controlled trial.

### Data extraction and statistical analysis

Both authors extracted data from the accepted articles independently without being blinded to authors, institutions, journal names and trial results. The extracted data included: number of restorations failures (n) and number of evaluated restorations (N) at the end of each follow-up period, per type of restorative treatment (HVGIC or amalgam) and cavity type (Class I or II). The n/N-data from each HVGIC study was statistically compared to that of each amalgam study and Odds ratios (OR) with 95% Confidence intervals (CIs) were computed using statistical software RevMan 4.1.2. The thus extracted and computed data was considered as the ‘test-data’ in this study.

The ‘control data’ was in turn extracted from a systematic review of 20 randomised control trials (RCTs) by the authors [34] that appraised the current clinical evidence regarding to the question as to whether, in patients with carious cavities, direct HVGIC restorations placed according to the atraumatic restorative treatment approach have a higher failure rate than conventional amalgam restorations (File S2). For the purpose of this study, only those RCTs were selected from the systematic review report that matched the uncontrolled clinical longitudinal studies according to investigated cavity type and follow-up period (Table 2).

The extracted data comprised of single dichotomous datasets per RCT, consisting of number of restorations failures (n) and number of evaluated restorations (N) for each cavity type at the end of each follow-up period.

The intention was to pool datasets of the same cavity type and follow-up period using random-effects meta-analysis (RevMan 4.1.2), if possible. The test results from uncontrolled clinical longitudinal studies were plotted together with the control results from the RCTs per follow-up period in two forest plots, for Class I and Class II restorations, separately. From the forest plots, trend directions were inferred from the overlap of confidence intervals and direction of point estimates; magnitudes of performance differences were inferred from the median point estimates (OR) with 25% and 75% percentile range, for both types of comparison. Mann-Whitney U test (Biostat 2009 software) was applied to test for statistically significant differences between the point estimates of both comparison types.

Alpha level for statistical significance was set at 5%.

## Results

### Extracted data and statistical analysis

**Naïve-indirect HVGIC/amalgam comparison of uncontrolled longitudinal data.** From the ten included HVGIC studies [18]–[25], [27], [28] seven n/N-datasets (DS 01-07) were extracted for Class I restorations after 12 months follow-up; eight (DS 08–13,25,26) after 24 months and three (DS 14–16) after 36 months, as well as five (DS 17–21) and three datasets (DS 22–24) for Class II restorations after 12 and 24 months, respectively.

From the two amalgam studies [29], [33] only one single n/N dataset could be extracted each for Class I after 12, 24 and 36 months, as well as for Class II after 12 and 24 months. Every HVGIC dataset was matched against the single n/N -amalgam dataset available for its cavity class and follow-up period. The 24 resulting (n/N – n/N) combined datasets are presented in Table 3.

**Table 3:** Extracted datasets from studies for analysis.

Follow-up period	Naïve indirect comparisons based on uncontrolled clinical longitudinal studies								Direct comparisons based on RCTs					
	DS	HVGIC				Amalgam			DS	Study	HVGIC		Amalgam	
		Study	n	N		Study	n	N			n	N	n	N
Class I cavities in permanent posterior teeth														
12 months	01	Lopez et al., 2005 [18]	8		39	Kiremitci and Bolay, 2003 [33]	0	32	RCT-1	Yip et al., 2002 [35]	0	21	0	22
	02	Zanata et al., 2011 [20]	2		146		0	32	RCT-2		0	17	0	22
	03	Ibiyemi et al., 2011 [21]	2		93		0	32	RCT-3	Frencken et al., 2006 [36]	33	487	33	403
	04	Barata et al., 2008 [22]	1		36		0	32						
	05	Ercan et al., 2009 [23]	5		21		0	32						
	06	Abid et al., 2002 [27]	15		126		0	32						
	07	Cefaly et al., 2007 [28]	1		16		0	32						
24 months	08	Lopez et al., 2005 [18]	8		29	Kiremitci and Bolay, 2003 [33]	2	32	RCT-4	Rahimtoola and van Amerongen, 2002 [37]	10	160	3	62
	09	Zanata et al., 2011 [20]	8		117		2	32	RCT-5	Frencken et al., 2006 [36]	22	397	34	323
	10	Ibiyemi et al., 2011 [21]	3		92		2	32						
	11	Barata et al., 2008 [22]	6		21		2	32						
	12	Ercan et al., 2009 [23]	1		18		2	32						
	13	Abid et al., 2002 [27]	42		111		2	32						
	25	Ziraps and Honkala, 2002 [24]*	2		27		2	32						
	26	Ziraps and Honkala, 2002 [24]**	1		18		2	32						
36 months	14	Gemert-Schriks et al., 2007 [19]	32		54	Kiremitci and Bolay, 2003 [33]	3	32	RCT-6	Frencken et al., 2006 [36]	14	348	9	267
	15	Wang et al., 2004 [25]	45		57		3	32						
	16	Abid et al., 2002 [27]	13		69		3	32						
Class II cavities in permanent posterior teeth														
12 months	17	Lopez et al., 2005 [18]	16	76		Sachdeo et al., 2004 [29]	0	35	RCT-7	Frencken et al., 2006 [36]	11	52	7	33
	18	Zanata et al., 2011 [20]	9	98			0	35						
	19	Barata et al., 2008 [22]	0	5			0	35						
	20	Ercan et al., 2009 [23]	11	17			0	35						
	21	Cefaly et al., 2007 [28]	1	13			0	35						
24 months	22	Lopez et al., 2005 [18]	19	53		Sachdeo et al., 2004 [29]	1	35	RCT-8	Frencken et al., 2006 [36]	3	34	10	23
	23	Zanata et al., 2011 [20]	27	62			1	35						
	24	Ercan et al., 2009 [23]	13	17			1	35						

DS  =  Datasets number; n  =  Number of failed restorations; N  =  Number of evaluated restorations; RCT  =  Randomised control trial;

HVGIC  =  High-viscosity glass-ionomer cement. *HVGIC  =  ChemFlex; **HVGIC  =  Fuji IX.

**Direct HVGIC/amalgam comparison of RCT data.** Eight (n/N – n/N) datasets from three RCTs [35]–[37] relevant to Class I and II restorations in posterior permanent teeth after 12, 24 and 36 months were extracted from Table 10 of the systematic review [34] (File S2).

**Figure 2:**
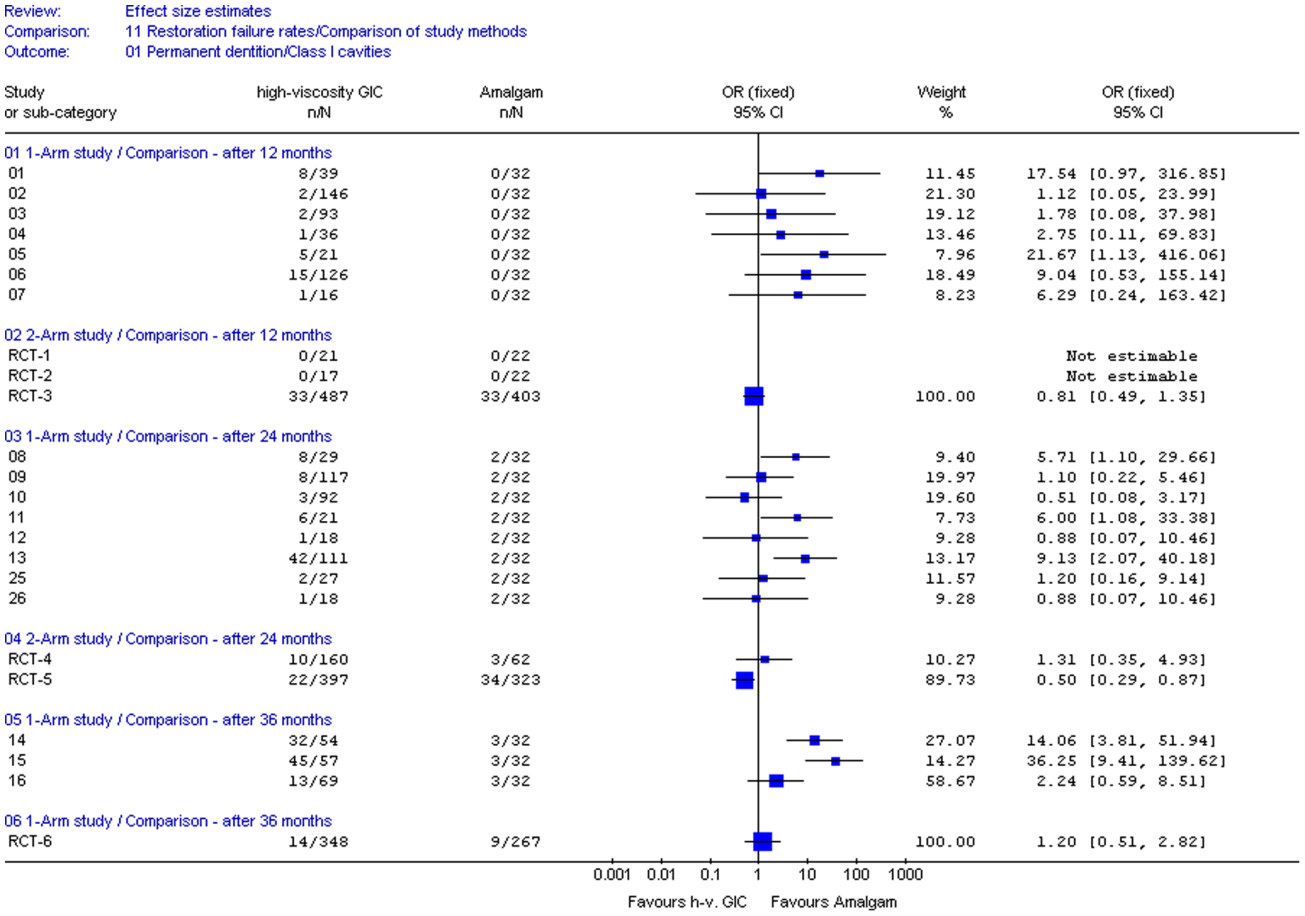
Computed effect estimates of studies: Failure rates of Class I restorations in posterior permanent teeth. OR  =  Odds ratio; CI  =  Confidence interval; n  =  Number of failed restorations; N  =  Number of evaluated restorations; h-v.  =  high-viscosity; GIC  =  Glass-ionomer cement; ‘1-arm study’  =  Naïve-indirect comparisons of uncontrolled longitudinal study; 2-arm study’  =  Direct comparison within a randomised control trial; Not estimable  =  Both interventions have essentially the same n/N data; i.e. OR  =  1.00; ‘Study or sub-category’  =  Dataset number of comparison (see Table 3).

**Figure 3:**
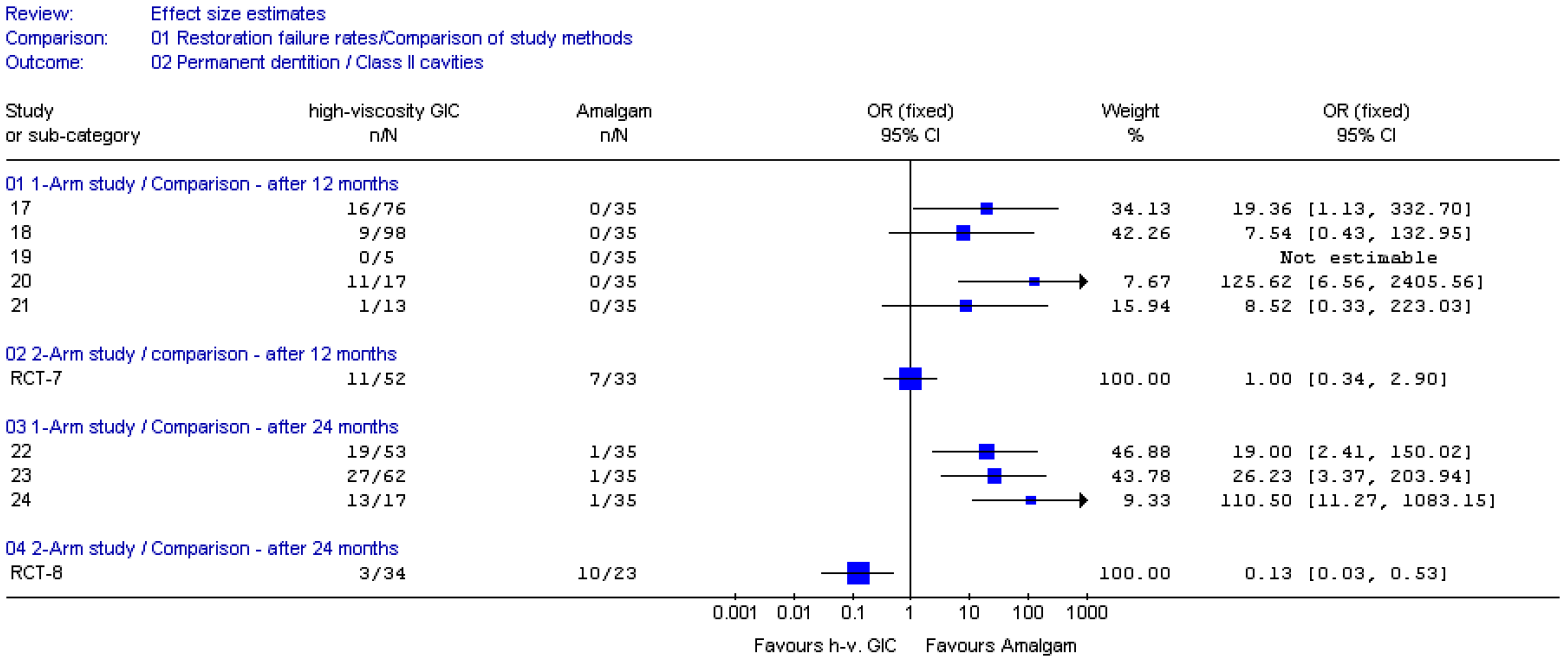
Computed effect estimates of studies: Failure rates of Class II restorations in posterior permanent teeth. OR  =  Odds ratio; CI  =  Confidence interval; n  =  Number of failed restorations; N  =  Number of evaluated restorations; h-v.  =  high-viscosity; GIC  =  Glass-ionomer cement; ‘1-arm study’  =  Naïve-indirect comparisons of uncontrolled longitudinal study; 2-arm study’  =  Direct comparison within a randomised control trial; Not estimable  =  Both interventions have essentially the same n/N data; i.e. OR  =  1.00; ‘Study or sub-category’  =  Dataset number of comparison (see Table 3).

The computed Odds ratios (95% CI) were plotted for Class I and II restorations and are shown in Figure 2 and 3, respectively. Meta-analysis of longitudinal study results was not conducted, as only one n/N dataset was available from amalgam studies per type of cavity and follow-up period against which all n/N datasets from HVGIC studies were set.

Figures 2 and 3 show that the width of the 95% confidence intervals (CI) differs largely between the two types of comparisons, which may be ascribed to the generally larger sample size in direct comparisons (RCTs). However, from the confidence intervals and point estimates (OR) the following could be observed:

- The 95% Confidence intervals from naïve-indirect comparisons generally overlap to the right side of the forest plots and the position of the point estimates on the plots is mainly situated to the right. Both factors suggest a trend direction favouring amalgam above HVGIC. The magnitude of the median point estimate: OR  =  6.29 (1.34 – 19.27) suggests a largely higher performance, in terms of a lower restoration failure rate, for amalgam.
- The 95% Confidence intervals from direct comparisons (RCT) overlap mainly to the left/centre of the forest plots and the point estimates on the plots are mostly situated towards the center. Both suggest an essentially similar trend for HVGIC and amalgam regarding their restoration failure rates. The median size of the point estimates (OR  =  1.00; 0.81 – 1.20) indicates similar to equal performance for the two types of restoration.

The distributions of the point estimates differed significantly for both comparison types (Mann–Whitney U  =  25, n_indirect_  =  26; n_direct_  =  8; p  =  0.0013, two-tailed).These results indicate that trends and performance differences inferred from naïve-indirect comparison based on evidence from uncontrolled clinical longitudinal studies and from direct comparisons based on RCT evidence do not have the same direction and magnitude. The null-hypothesis was therefore rejected.

## Discussion

### Limitations of study method

The aim of this empirical study was to investigate whether trends and performance differences between conventional amalgam and direct HVGIC restorations in posterior teeth can be inferred through naïve-indirect comparison of failure rates from uncontrolled clinical longitudinal studies. The objective was to test the null-hypothesis that trends and performance differences inferred from naïve-indirect comparison based on evidence from uncontrolled clinical longitudinal studies and from direct comparisons based on RCT evidence have similar directions and magnitude.

The intention was to pool datasets of the same cavity type and follow-up period using random-effects meta-analysis (RevMan 4.1.2), if possible. However, meta-analysis of longitudinal study results was not conducted, as only one n/N dataset was available from amalgam studies per type of cavity and follow-up period against which all n/N datasets from HVGIC studies were set. Pooling of these results would have generated erroneously too narrow confidence intervals and thus potentially misleading summary outcomes.

Data was drawn only from studies published in English. The reason for this language restriction was the consideration that the inclusion of non-English trials may have had little effect on summary treatment effect estimates but may rather be assumed as confirmatory [38], [39]. Only uncontrolled longitudinal studies that were listed in PubMed/Medline from 2002 were searched, in order to limit the risk of any possible chronological bias, as no RCTs that provided a direct comparison between HVGIC and amalgam restorations before that date could be identified [34]. Further focus was on HVGIC studies that placed tooth restorations using the atraumatic restorative treatment (ART) approach. The reason was that the RCT data were drawn exclusively from a systematic review [34] that included HVGIC/ART restorations and this ensured that the studies of both, uncontrolled longitudinal design and RCT did not differ in this point. However, in the literature search we did not identify any HVGIC longitudinal studies that were not based on ART.

The restrictions of this study may have limited the data available. However, the authors are confident that the identified cohort of studies represents the clinical evidence from most, if not all, clinical longitudinal studies and RCTs relevant to posterior HVGIC and amalgam restorations in the permanent dentition that have been listed in PubMed/Medline during the 2002–2012 period.

### Study results

The results of this investigation suggest that the trend direction and magnitude of performance differences inferred from study results are highly affected by the utilized type of comparison and type of study design (i.e. naïve-indirect comparison of uncontrolled longitudinal evidence versus direct comparison within RCTs). The results from naïve-indirect comparison of uncontrolled longitudinal evidence are in keeping with the current general consensus on clinical HVGIC merits and is also expressed in the DGZ/DGZMK statement of Germany [14].

From each comparison type, different trend directions and magnitudes of performance differences can be inferred (i.e. the failure rate of HVGIC being inferior/equal to that of conventional amalgam restorations in permanent posterior teeth). The null-hypothesis was rejected. This raises questions regarding the reliability of the study designs and comparison methods for subsequent inference:

Randomised control trials (RCT) are 2- or more arm studies where the different intervention groups have been formed through random allocation. RCTs have been recognised as the ‘gold-standard’ in clinical trial methodology [40].

Clinical uncontrolled longitudinal studies are defined as a subset of non-RCTs without use of a comparison group, which evaluate the effect of a particular treatment in patients who are all offered this same particular treatment [41]. The rationale of this study type comprises of: (a) application of a pre-test measurement to a single group of patients, e.g. ‘count’ (absence) of restoration failures after restoration placement at baseline; (b) reapplication of the same measurement (count of restoration failures) as post-test after a certain time period; e.g. after 12, 24 or 36 months [42]. Clinical uncontrolled longitudinal studies have been found to be more efficient than cross-sectional studies in estimating the average change of measurement and its variation between individual patients [43]. They are very common in medicine [44], are faster, more convenient and less expensive to conduct than RCTs [41] and function as valuable pilot studies for guiding the planning of subsequent RCTs, e.g. in the estimation of effect sizes as basis for RCT sample size calculation [41], [45].

However, despite it’s stated merits the rationale of uncontrolled longitudinal studies carries the logical “*post hoc ergo propter hoc*” or ‘false cause’ fallacy [46] as its results suggest that a causal relationship exists between the applied intervention (e.g. the type of the restorative material) and the observed average change of post-test measurement (e.g. the restoration failure rate). Such erroneously assumed causality does not take into account other potential factors that may have caused or at least influenced the post-test measurement, which the uncontrolled longitudinal study design is unable to exclude.

Owing to the lack of a randomly selected comparison group, uncontrolled longitudinal studies are vulnerable to many sources of invalidity that are often difficult to rule out [42]. These include external confounding factors that can be known or unknown to both patient and study operator and whose effects may increase with length of follow-up period [42]. Another source of vulnerability is regression to the mean, either due to variations within patients or to measurement errors that cannot be corrected, due to lack of a control group [41], [47]. In addition, uncontrolled longitudinal studies are at higher risk of investigator bias and are thus more likely to lead to statistically significant results favouring one type of treatment above another [41]. Because of these shortcomings, uncontrolled longitudinal studies provide weak evidence and their results should thus not be used to guide clinical practice [45], [48].

The shortcomings of the uncontrolled longitudinal study design have further impact when its results are used for naïve-indirect comparisons between two competing clinical interventions. Such comparisons are based on the assumptions of homogeneity, similarity and consistency between uncontrolled longevity studies as their data sources. Moreover, study characteristics that are not based on randomised distribution of variables within one single clinical/methodological setting cannot assure the certainty of such assumptions. Consequently, investigations have established that results from naïve-indirect comparisons have an inflated probability of statistical significance with a 30% smaller standard error (SE) than direct comparisons based on randomised control trials [16]. It was further found that 40% of confidence intervals generated from naïve-indirect comparisons do not contain the correct effect size value and that results of naïve-indirect comparisons have very poor agreement with results from direct comparisons (kappa  =  0.28). The reasons for such discrepancies have been mainly ascribed to lack of compatibility, due to different prognostic factors, between patients from the different studies included in naïve-indirect comparisons accompanied by risk of random error (5% chance of type I error), that may cause a statistical significance even if the null-hypothesis is true [16]. The results of the present study, particularly the statistically higher median point estimate established from naïve-indirect comparison in favour of amalgam above HVGIC (OR 6.29; 1.34 – 19.27), appear to be in line with such observations.

Despite the more promising trends and performance differences that can be inferred from direct comparisons within RCTs regarding the failure rate of direct posterior HVGIC restorations in permanent teeth, shortcomings in the current evidence remain (e.g. related to aspects of internal validity and sample size). These require further research [49]. Nevertheless, these shortcomings do not provide evidence in support of the recommendation that HVGIC are not suitable for use as permanent posterior tooth restoration materials [14], which only direct comparisons within RCTs can provide. In this context it is interesting to note that despite a broad systematic literature appraisal no summary RCT evidence could be established in support of the hypothesis that “direct HVGIC restorations are inferior to those of amalgam in posterior cavities of permanent teeth” [34], [50].

It is recommended that any guidance for clinical practice should be based on direct comparisons from randomised control trials, ideally appraised during systematic reviews of the clinical literature. Where RCT evidence has not as yet been established in clinical fields, clinical guidance should at least avoid recommendations based on flawed data comparisons (i.e. naïve-indirect comparison) and fallacious study methodology (i.e. uncontrolled longitudinal study design) that carry high risk of confounding and systematic error.

## Conclusions

The results of this study indicate that differences concerning the failure rate of direct HVGIC versus amalgam restorations, inferred from naïve-indirect comparison and from direct comparisons based on RCT evidence are not similar in direction and magnitude. The discrepancy is ascribed to severe shortcomings in uncontrolled longitudinal clinical study design and the flawed method of naïve-indirect comparison. Both are found to carry high confounder influence risk and bias/systematic error and so may have inflated its results favouring amalgam above HVGIC restorations. Specifically, the naïve-indirect comparison of clinical characteristics of high-viscosity glass ionomer cements against gold standards for posterior permanent restorations, such as conventional amalgam fillings, based on uncontrolled clinical longitudinal studies may have augmented the reasons for the current negative clinical recommendations for HVGICs as, for example expressed in the DGZ/DGZMK statement of Germany. The reliance of such directives on naïve-indirect comparison based on uncontrolled clinical longitudinal study evidence calls for attention and revision.

## Supporting Information

File S1 **Reference analysis: DGZ/DGZMK statement.**
(XLS)

File S2 **Methodological and clinical characteristics of Randomised Control Trials.**
(DOC)

Checklist S1 **PRISMA 2009 Checklist.**
(DOC)

## References

[1] Frencken JE, Van't Hof MA, Van Amerongen WE, Holmgren CJ (2004) Effectiveness of single-surface ART restorations in the permanent dentition: a meta-analysis. J Dent Res 83: 120–123. 14742648 10.1177/154405910408300207

[2] Yoshida Y, Van Meerbeek B, Nakayama Y, Snauwaert J, Hellemans L, et al (2000) Evidence of chemical bonding at biomaterial-hard tissue interfaces. J Dent Res 79: 709–771. 10728971 10.1177/00220345000790020301

[3] Mickenautsch S, Yengopal V, Leal SC, Oliveira LB, Bezerra AC, et al (2009) Absence of carious lesions at margins of glass-ionomer and amalgam restorations: a meta- analysis. Eur J Paediatr Dent 10: 41–46. 19364244

[4] Ericson D, Kidd EAM, McComb D, Mjor I, Noack MJ (2003) Minimally invasive dentistry – concept and techniques in cariology. Oral Health Prev Dent 1: 59–72. 15643750

[5] Fuks AB (2002) The use of amalgam in pediatric dentistry. Pediatr Dent 24: 448–455. 12412959

[6] Carvalho CA, Fagundes TC, Barata TJ, Ferrari M, Navarro MF (2007) Influence of ultrasonic setting on microhardness of glass-ionomer cements. Int Dent SA 9: 24–32.

[7] Torabzadeh H, Ghasemi A, Shakeri S, Alireza Baghban AA, Razmavar S (2011) Effect of powder/liquid ratio of glass ionomer cements on flexural and shear bond strengths to dentin. Braz J Oral Sci 10: 204–207.

[8] Piwowarczyk A, Ottl P, Lauer HC (2001) Laboratory strength of glass ionomer and zinc phosphate cements. J Prosthodont 10: 140–147. 11641841 10.1111/j.1532-849x.2001.00140.x

[9] Zahra VN, Kohen SG, Macchi R (2011) Powder-liquid ratio and properties of two restorative glass ionomer cements. Acta Odontol Latinoam 24: 200–204. 22165320

[10] Busanello L, Telles M, Miranda WG Jr, Imparato JC, Jacques LB, et al (2009) Compressive strength of glass ionomer cements used for atraumatic restorative treatment. Rev Odonto Ciênc 24: 295–298.

[11] Bresciani E, Barata Tde J, Fagundes TC, Adachi A, Terrin MM, et al (2004) Compressive and diametral tensile strength of glass ionomer cements. J Appl Oral Sci 12: 344–348. 20976409 10.1590/s1678-77572004000400017

[12] Heintze SD (2007) Systematic reviews: I. The correlation between laboratory tests on marginal quality and bond strength. II. The correlation between marginal quality and clinical outcome. J Adhes Dent 9 Suppl 177–106. 18341236

[13] Heintze SD, Zimmerli B (2011) Relevance of in vitro tests of adhesive and composite dental materials. A review in 3 parts. Part 3: in vitro tests of adhesive systems. Schweiz Monatsschr Zahnmed 121: 1024–1040. 22139713

[14] DGZ/DGZMK (2005) [Direkte Kompositrestaurationen im Seitenzahnbereich – Indikation und Lebensdauer] (Document in German). Available: http://www.dgzmk.de/uploads/tx_szdgzmkdocuments/ Kompositrestaurationen im_Seitenzahnbereich.pdf. Accessed 2012 Sep 29.

[15] Manhart J, Chen H, Hamm G, Hickel R (2004) Buonocore Memorial Lecture. Review of the clinical survival of direct and indirect restorations in posterior teeth of the permanent dentition. Oper Dent 29: 481–508. 15470871

[16] Glenny AM, Altman DG, Song F, Sakarovitch C, Deeks JJ, et al (2005) Indirect comparisons of competing interventions. Health Technol Assess 9: 1–134. 10.3310/hta9260 16014203

[17] Ibiyemi O, Bankole OO, Oke GA (2011) Survival rates of two atraumatic restorative treatment (ART) types in occlusal carious permanent teeth after two years. Afr J Med Med Sci 40: 127–134. 22195380

[18] Lopez N, Simpser-Rafalin S, Berthold P (2005) Atraumatic restorative treatment for prevention and treatment of caries in an underserved community. Am J Public Health 95: 1338–1339. 16006415 10.2105/AJPH.2004.056945 PMC1449363

[19] van Gemert-Schriks MC, van Amerongen WE, ten Cate JM, Aartman IH (2007) Three-year survival of single- and two-surface ART restorations in a high-caries child population. Clin Oral Investig 11: 337–343. 10.1007/s00784-007-0138-8 PMC2099161 17710452

[20] Zanata RL, Fagundes TC, Freitas MC, Lauris JR, Navarro MF (2011) Ten-year survival of ART restorations in permanent posterior teeth. Clin Oral Investig 15: 265–271. 10.1007/s00784-009-0378-x PMC3055991 20140470

[21] Ibiyemi O, Bankole OO, Oke GA (2011) Assessment of Atraumatic Restorative Treatment (ART) on the permanent dentition in a primary care setting in Nigeria. Int Dent J 61: 2–6. 10.1111/j.1875-595X.2011.00001.x PMC9374807 21382026

[22] Barata TJ, Bresciani E, Mattos MC, Lauris JR, Ericson D, et al (2008) Comparison of two minimally invasive methods on the longevity of glass ionomer cement restorations: short-term results of a pilot study. J Appl Oral Sci 16: 155–160. 19089209 10.1590/S1678-77572008000200014 PMC4327637

[23] Ercan E, Dülgergil CT, Soyman M, Dalli M, Yildirim I (2009) A field-trial of two restorative materials used with atraumatic restorative treatment in rural Turkey: 24-month results. J Appl Oral Sci 17: 307–314. 19668990 10.1590/S1678-77572009000400008 PMC4327647

[24] Ziraps A, Honkala E (2002) Clinical trial of a new glass ionomer for an atraumatic restorative treatment technique in class I restorations placed in Latvian school children. Med Princ Pract 11 Suppl 144–47. 12123116 10.1159/000057778

[25] Wang L, Lopes LG, Bresciani E, Lauris JR, Mondelli RF, et al (2004) Evaluation of Class I ART restorations in Brazilian schoolchildren: three-year results. Spec Care Dentist 24: 28–33. 15157057 10.1111/j.1754-4505.2004.tb01676.x

[26] Lo EC, Holmgren CJ, Hu D, van Palenstein Helderman W (2007) Six-year follow up of atraumatic restorative treatment restorations placed in Chinese school children. Community Dent Oral Epidemiol 35: 387–392. 17822487 10.1111/j.1600-0528.2006.00342.x

[27] Abid A, Chkir F, Ben Salem K, Argoubi K, Sfar-Gandoura M (2002) Atraumatic restorative treatment and glass ionomer sealants in Tunisian children: survival after 3 years. East Mediterr Health J 8: 315–323. 15339119

[28] Cefaly DF, Barata TJ, Bresciani E, Fagundes TC, Lauris JR, et al (2007) Clinical evaluation of multiple-surface ART restorations: 12 month follow-up. J Dent Child (Chic) 74: 203–208. 18482515

[29] Sachdeo A, Gray GB, Sulieman MA, Jagger DC (2004) Comparison of wear and clinical performance between amalgam, composite and open sandwich restorations: 2-year results. Eur J Prosthodont Restor Dent 12: 15–20. 15058177

[30] Soncini JA, Maserejian NN, Trachtenberg F, Tavares M, Hayes C (2007) The longevity of amalgam versus compomer/composite restorations in posterior primary and permanent teeth: findings From the New England Children's Amalgam Trial. J Am Dent Assoc 138: 763–772. 17545265 10.14219/jada.archive.2007.0264

[31] Bernardo M, Luis H, Martin MD, Leroux BG, Rue T, et al (2007) Survival and reasons for failure of amalgam versus composite posterior restorations placed in a randomized clinical trial. J Am Dent Assoc 138: 775–783. 17545266 10.14219/jada.archive.2007.0265

[32] Van Nieuwenhuysen JP, D'Hoore W, Carvalho J, Qvist V (2003) Long-term evaluation of extensive restorations in permanent teeth. J Dent 31: 395–405. 12878022 10.1016/s0300-5712(03)00084-8

[33] Kiremitci A, Bolay S (2003) A 3-year clinical evaluation of a gallium restorative alloy. J Oral Rehabil 30: 664–667. 12787466 10.1046/j.1365-2842.2003.01007.x

[34] Mickenautsch S, Yengopal V (2012) Failure rate of high-viscosity GIC based ART compared to that of conventional amalgam restorations - evidence from a systematic review update. S Afr Dent J 67: 329–331. 23951787

[35] Yip KH, Smales RJ, Gao W, Peng D (2002) The effects of two cavity preparation methods on the longevity of glass ionomer cement restorations: an evaluation after 12 months. J Am Dent Assoc 133: 744–751. 12085859 10.14219/jada.archive.2002.0272

[36] Frencken JE, Taifour D, van 't Hof MA (2006) Survival of ART and amalgam restorations in permanent teeth of children after 6.3 years. J Dent Res 85: 622–666. 16798862 10.1177/154405910608500708

[37] Rahimtoola S, van Amerongen E (2002) Comparison of two tooth-saving preparation techniques for one-surface cavities. ASDC J Dent Child 69: 16–26. 12119808

[38] Jüni P, Holenstein F, Sterne J, Bartlett C, Egger M (2002) Direction and impact of language bias in meta-analyses of controlled trials: empirical study. Int J Epidemiol 31: 115–123. 11914306 10.1093/ije/31.1.115

[39] Moher D, Pham B, Klassen TP, Schulz KF, Berlin JA, et al (2000) What contributions do languages other than English make on the results of meta-analyses? J Clin Epidemiol 53: 964–972. 11004423 10.1016/s0895-4356(00)00188-8

[40] Pocock SJ (1983) Clinical trials: a practical approach. Chichester: Wiley.

[41] Wang D, Bakhai A (2006) Clinical Trials - A Practical Guide to Design, Analysis, and Reporting. Remedica: London.

[42] Huitema BE (2011) Uncontrolled Clinical Trials. In: The Analysis of Covariance and Alternatives: Statistical Methods for Experiments, Quasi-Experiments, and Single-Case Studies, 2^nd^ Edition; John Willey and Sons Inc, pp. 609.

[43] Reed RB (1967) The rationale of longitudinal studies. J Dent Res 46: 1208. 5235009 10.1177/00220345670460061401

[44] Arabi Y (2005) Study Designs in healthcare research. Saudi Med J 26: 1175–1179. 16127508

[45] White A, Ernst E (2001) The case for uncontrolled clinical trials: a starting point for the evidence base for CAM. Compl Therapies Med 9: 111–115. 10.1054/ctim.2001.0441 11444891

[46] Türp JC, Schwarzer G (2003) The effectiveness of therapeutic measures: the post-hoc-ergo-propter-hoc fallacy. (Article in German). Schweiz Monatsschr Zahnmed 113: 36–46. 12602200

[47] James KE (1973) Regression toward the mean in uncontrolled clinical studies. Biometrics 29: 121–130. 4570667

[48] Sutherland SE: Evidence-based dentistry (2001) Part IV. Research design and levels of evidence. J Can Dent Assoc 67: 375–378. 11468093

[49] Mickenautsch S (2012) Research gaps identified during systematic reviews of clinical trials: Glass-ionomer cements. BMC Oral Health 12: 18. 22747674 10.1186/1472-6831-12-18 PMC3461440

[50] Wang X, Nie J, Cai X, Yengopal V, Mickenautsch S (2012) Failure rate of atraumatic restorative treatment using high-viscosity glass-ionomer cement compared to conventional amalgam restorative treatment in primary and permanent teeth: a systematic review of Chinese trials. J Minim Interv Dent 5: : 377 – 415.

