# Supplementary material for: Direct Contra Naïve-Indirect Comparison of Clinical Failure Rates between High-Viscosity GIC and Conventional Amalgam Restorations: An Empirical Study
Source: PLoS One. 2013 Oct 28;8(10):e78397. doi: 10.1371/journal.pone.0078397 (PMC3810255; doi:10.1371/journal.pone.0078397)
Supplement: File S2 — Methodological and clinical characteristics of Randomised Control Trials. (DOC) [file pone.0078397.s002.doc]

**Direct contra naïve-indirect comparison of clinical failure rates between high-viscosity GIC and conventional amalgam restorations**

**Mickenautsch S, Yengopal V**

**SYSTEM Initiative/Department of Community Dentistry, Faculty of Health Sciences, University of the Witwatersrand - 7 York Rd., Parktown/Johannesburg 2193, South Africa**

**APPENDIX FILE 2**

**(Reference numbers in the tables are not identical with that of main article and only refer to the reference list beneath the tables)**

Methodological and clinical characteristics of Randomised Control Trials – Part 1

| 1^st^ Author | Year | Reference | Place of  Trial | Participants | | | Study design | ART cavity  conditioned |
| --- | --- | --- | --- | --- | --- | --- | --- | --- |
|  |  |  |  | Age | Gender | Characteristics |  |  |
| Chen | 2006 | [1] | Liaocheng | mean 9.7 yrs ART group; mean 9.6 yrs amalgam group | ART group:  74 m; 46 f  Amalgam group:  65 m; 55 f | Children with occlusal or buccal fissure caries into dentin; for each patient 2 teeth were selected; cavity single or small multiple surface large enough for smallest excavator to enter; no pulp symptoms or periodontal disease; tooth symptom free; parental and patient consent. | PG |  |
|  |  |  |  |  |  |  | PG |  |
|  |  |  |  |  |  |  | PG |  |
| Chen(b) | 2001 | [2] | Shenyang | 4-8 yrs |  | Children with caries on both sides of his/her (mouth) | SM |  |
|  |  |  |  |  |  |  |  |  |
| She | 2003 | [3] | Sichuan | mean 6.72 yrs |  | Children with one or more teeth with dentin caries; cavity single or small multiple surface large enough for smallest excavator to enter; no pulp symptoms | PG |  |
|  |  |  |  |  |  |  | PG |  |
| Wang | 2004 | [4] | Taizhou |  | 108 m; 92 f | Cavity single or small multiple surface large enough for smallest excavator to enter; no pulp symptoms | PG |  |
|  |  |  |  |  |  |  |  |  |
| Li(b) | 2005 | [5] | Jiangmen | 60-78 yrs | 98 m; 106 f |  | PG |  |
|  |  |  |  |  |  |  | PG |  |
| Ling | 2003 | [6] | Wuxi | 6-8 yrs | 53 m; 53 f | Shallow or intermediate carious lesions on both sites of the mouth; no pulp symptoms or periodontal disease; cavity size less than 2/3 of occlusal surface | SM |  |
|  |  |  |  |  |  |  |  |  |
| Qiu | 2007 | [7] | Dongying |  |  | One or more carious lesion into dentin; cavity single or small multiple surface large enough for smallest excavator to enter; no pulp symptoms or periodontal disease; tooth symptom free | PG |  |
|  |  |  |  |  |  |  | PG |  |
| Li | 2002 | [8] | Chengdu | 6-8 yrs, mean 608 yrs |  | One or more carious lesion into dentin; cavity single or small multiple surface large enough for smallest excavator to enter; no pulp symptoms or periodontal disease; tooth symptom free | PG |  |
|  |  |  |  |  |  |  | PG |  |
| Miranda | 2005 | [9] | Rio de J | 3-9 yrs, mean 5.71, SD 1.77 | 47 m; 33 f | 2 teeth with carious lesions into dentin; healthy pulp; access > 1mm; HEX accessible  oral hygiene: 2x tooth brushing/day | SM | Yes |
|  |  |  |  |  |  |  | SM | Yes |
| Taifour | 2002 | [10] | Damascus | 6-7 yrs |  | Dental lesion with opening diameter >0.9mm; healthy pulp; dmfts/t molars 9.0/4.4 | PG | Yes |
|  |  |  |  |  |  |  | PG | Yes |
| Yu | 2004 | [11] | Beijing | mean 7.4 yrs, SD 1.24 | 27m; 33f | Healthy children with at least 1 pair of carious primary molars; | SM | (Yes) |
|  |  |  |  |  |  |  | SM | (Yes) |
|  |  |  |  |  |  |  | SM | (Yes) |
|  |  |  |  |  |  |  | SM | (Yes) |
| Honkala | 2003 | [12] | Kuwait | 2-9 yrs; mean 5.7 yrs | 12m; 6f | At least one carious lesion in both sides of the jaw; teeth symptom/pain free;  uncooperative; high-caries risk | SM |  |
|  |  |  |  |  |  |  | SM |  |
| Yip | 2002 | [13] | Beijing | mean 34.6 yrs, SD 12.3 | 26m; 42f | Volunteer adult patients with at least 2 small occlusal lesions with opposing tooth contact | SM | Yes |
|  |  |  |  |  |  |  | SM | Yes |
| Rahimtoola | 2002 | [14] | Pakistan | 6-16 yrs, mean 11.4 |  | DMFT ART/AM group: 3.15/2.98 | SM | Yes |
| Frencken | 2006 | [15] | Damascus | 6-9 yrs | 287m; 310f | Dental lesion with opening diameter >0.9mm; DMFS/T 1.6/1.4; healthy pulp;  no criteria set for cavity size | PG | Yes |
|  |  |  |  |  |  |  | PG | Yes |
|  |  |  |  |  |  |  | PG | Yes |
|  |  |  |  |  |  |  | PG | Yes |
|  |  |  |  |  |  |  | PG | Yes |
|  |  |  |  |  |  |  | PG | Yes |
|  |  |  |  |  |  |  | PG | Yes |
|  |  |  |  |  |  |  | PG | Yes |
|  |  |  |  |  |  |  | PG | Yes |
|  |  |  |  |  |  |  | PG | Yes |
| Yip | 2002 | [16] | Beijing | 7-9 yrs |  |  | SM | (Yes) |
|  |  |  |  |  |  |  | SM | (Yes) |
| Gao | 2003 | [17] | Beijing | Mean 37.7 yrs; SD 15.1 | 4m; 13f | Volunteer adult patients with at least 2 small occlusal lesions with opposing tooth contact | SM | Yes |
| Estupiñán-Day | 2006 | [18] | Ecuador | 7-9 yrs | 42%m, 58%f | At least 1 tooth with initial enamel or dentinal lesion; not close to pulp; no pulp symptoms | PG |  |
|  |  |  | Panama |  | 50%m, 50%f |  | PG |  |
|  |  |  | Uruguay |  | 51%m, 49%f |  | PG |  |
|  |  |  | Ecuador |  | 42%m, 58%f |  | PG |  |
|  |  |  | Panama |  | 50%m, 50%f |  | PG |  |
|  |  |  | Uruguay |  | 51%m, 49%f |  | PG |  |
| Ye | 2006 | [19] | Guangdong | 3-4 yrs | 73m; 65f | Cavity single surface large enough for smallest excavator to enter; no pulp symptoms or periodontal disease; tooth symptom free | PG |  |
| Ling | 2003 | [20] | (China) | 6-8 yrs | 53m; 53f | Cavity single surface large enough for smallest excavator to enter; no pulp symptoms or periodontal disease; tooth symptom free | PG |  |
|  |  |  |  |  |  |  |  |  |

Methodological and clinical characteristics of Randomised Control Trials – Part 2

| 1^st^ Author | Year | Ref. | ART group  GIC | Dentition | Tooth | Cavity | BSL (n)  Participants | BSL (n)  Teeth | Evaluation  Method | Criteria | N | n | Follow-up  period |
| --- | --- | --- | --- | --- | --- | --- | --- | --- | --- | --- | --- | --- | --- |
|  |  |  |  |  |  |  |  |  |  |  |  |  |  |
| Chen | 2006 | [1] |  | primary |  | 1SF/>1SF | 120 | 240 | Clinical | ART  Mod.1 | 231 | 24 | 1 yr |
|  |  |  |  |  |  |  | 120 | 240 |  |  | 225 | 55 | 2 yrs |
|  |  |  |  |  |  |  | 120 | 240 |  |  | 215 | 57 | 3 yrs |
| Chen(b) | 2001 | [2] | ‘FX glass ionomer’  by a Matsukaze Co Ltd Japan | primary | molar | 1SF | 104 |  | Clinical | Failure: Marginal gap between restoration and tooth; caries; partly or completely lost; pain | 52 | 5 | 1 yr |
|  |  |  |  |  |  |  |  |  |  |  | 21 | 3 | 2 yrs |
| She | 2003 | [3] | ‘glass ionomer  (Dentsply)’ | primary |  | 1SF/>1SF | 84 | 154 | Clinical | ART  Mod.2 | 135 | 9 | 1 yr |
|  |  |  |  |  |  |  | 84 | 154 |  |  | 123 | 22 | 2 yrs |
| Wang | 2004 | [4] | ‘FX glass ionomer’  by a Matsukaze Co Ltd Japan | primary | molar | 1SF/>1SF | 100 | 165 | Clinical | Failure: Marginal gap between restoration and tooth; need for repair; partly or completely lost; | 160 | 28 | 1 yr |
|  |  |  |  |  |  |  |  |  |  |  | 157 | 49 | 2 yrs |
| Li(b) | 2005 | [5] | Fuji IX | permanent |  | Class I |  |  | Clinical | Failure: Pain; Marginal gap between restoration and tooth; marginal discoloration; partly or completely lost; caries | 54 | 15 | 2 yrs |
|  |  |  |  |  |  | Class II |  |  |  |  | 72 | 37 | 2 yrs |
| Ling | 2003 | [6] | ‘FX glass ionomer’  by a Matsukaze Co Ltd Japan | primary | molar |  | 106 | 106 | Clinical | ART  Mod.3 | 106 | 16 | 1 yr |
|  |  |  |  |  |  |  | 106 | 106 |  |  | 106 | 23 | 2 yrs |
| Qiu | 2007 | [7] | Ketac Molar | primary |  | 1SF/>1SF | 70 | 198 | Clinical | ART  Mod.4 | 182 | 10 | 1 yr |
|  |  |  |  |  |  |  | 70 | 198 |  |  | 168 | 23 | 2 yrs |
| Li | 2002 | [8] | ‘glass ionomer (Dentsply)’ | primary |  | 1SF/>1SF | 128 | 154 | Clinical | ART  Mod.5 | 132 | 9 | 1 yr |
|  |  |  |  |  |  |  | 128 | 154 |  |  | 118 | 22 | 2 yrs |
| Miranda | 2005 | [9] | Ketac Molar | primary | molar | 1SF |  | 36 | Clin/X-Ray | ARTC | 33 | 1 | 1 yr |
|  |  |  |  |  |  | >1SF |  | 44 |  |  | 35 | 7 | 1 yr |
| Taifour | 2002 | [10] | Ketac Molar/Fuji IX | primary | molar | 1SF |  | 476 | Clinical | ARTC | 388 | 54 | 3 yrs |
|  |  |  |  |  |  | >1SF |  | 610 |  |  | 487 | 250 | 3 yrs |
| Yu | 2004 | [11] | Fuji IX | primary | molar | Class I |  | 19 | Clinical | ARTC | 18 | 1 | 1 yr |
|  |  |  | Ketac Molar |  |  |  |  | 16 |  |  | 13 | 1 | 1 yr |
|  |  |  | Fuji IX |  |  |  |  | 17 |  |  | 6 | 1 | 2 yrs |
|  |  |  | Ketac Molar |  |  |  |  | 12 |  |  | 5 | 0 | 2 yrs |
| Honkala | 2003 | [12] | ChemFlex | primary | molar | Class I | 18 |  | Clinical | ARTC | 26 | 2 | 22 mo |
|  |  |  |  |  |  | Class II | 18 |  |  |  | 9 | 1 | 22 mo |
| Yip* | 2002 | [13] | Fuji IX | permanent | molar | Class I |  | 29 | Clin/Indirect | USPHS | 21 | 0 | 1 yr |
|  |  |  | Ketac Molar |  |  |  |  | 26 | Clinical |  | 17 | 0 | 1 yr |
| Rahimtoola | 2002 | [14] | Fuji IX | permanent |  | (1SF) |  |  | Clinical | ARTC | 160 | 10 | 2 yrs |
| Frencken | 2006 | [15] | Ketac Molar/Fuji IX | permanent |  | 1SF | 302 | 487 | Clinical | ARTC | 487 | 33 | 1.3 yrs |
|  |  |  |  |  |  |  | 254 | 487 |  |  | 397 | 22 | 2.3 yrs |
|  |  |  |  |  |  |  | 224 | 397 |  |  | 348 | 14 | 3.3 yrs |
|  |  |  |  |  |  |  | 193 | 348 |  |  | 288 | 14 | 4.3 yrs |
|  |  |  |  |  |  |  | 102 | 288 |  |  | 161 | 8 | 5.3 yrs |
|  |  |  |  |  |  |  | 98 | 161 |  |  | 153 | 15 | 6.3 yrs |
|  |  |  |  |  |  | >1SF | 28 | 52 |  |  | 52 | 11 | 1.3 yrs |
|  |  |  |  |  |  |  | 20 | 52 |  |  | 34 | 3 | 2.3 yrs |
|  |  |  |  |  |  |  | 18 | 34 |  |  | 29 | 4 | 3.3 yrs |
|  |  |  |  |  |  |  | 15 | 29 |  |  | 21 | 3 | 4.3 yrs |
| Yip | 2002 | [16] | Fuji IX | primary | molar | Class I |  |  | Clinical | ARTC | 20 | 1 | 1 yr |
|  |  |  | Ketac Molar |  |  |  |  |  |  |  | 17 | 1 | 1 yr |
| Gao* | 2003 | [17] | Ketac Molar/Fuji IX | permanent | molar | Class I |  | 55 | Clinical | USPHS | 17 | 1 | 30 mo |
| Estupiñán-Day | 2006 | [18] |  | permanent | molar |  |  | 1336 |  |  | 1206 | 127 | 1 yr |
|  |  |  |  |  |  |  |  | 769 |  |  | 672 | 14 | 1 yr |
|  |  |  |  |  |  |  |  | 341 |  |  | 301 | 12 | 1 yr |
|  |  |  |  |  |  |  |  | 1336 |  |  | 1003 | 168 | 2 yrs |
|  |  |  |  |  |  |  |  | 769 |  |  | 625 | 22 | 2 yrs |
|  |  |  |  |  |  |  |  | 341 |  |  | 304 | 19 | 2 yrs |
| Ye | 2006 | [19] | Fuji IX | primary | molar | 1SF/>1SF | 70 | 186 | Clinical | ART /Mod.6 | 180 | 30 | 1 yr |
| Ling | 2003 | [20] | ‘FX glass ionomer’  by a Matsukaze Co Ltd Japan | primary | molar | Class I |  |  | Clinical | Success: Filling complete, slight defect on margin <0.5 mm; Failure: Marginal defect >0.5 mm, fracture on tooth or filling, filling missing, other treatment performed, tooth lost | 106 | 16 | 1 yr |
|  |  |  |  |  | molar |  |  |  |  |  | 106 | 23 | 2 yrs |

*Same clinical trial

Methodological and clinical characteristics of Randomised Control Trials – Part 3

| 1^st^ Author | Year | Ref. | Amalgam group | | Tooth | Cavity | BSL (n)  Participants | BSL (n)  Teeth | Evaluation  Method | Criteria | N | n | Follow-up  period |
| --- | --- | --- | --- | --- | --- | --- | --- | --- | --- | --- | --- | --- | --- |
|  |  |  | AM | Dentition |  |  |  |  |  |  |  |  |  |
| Chen | 2006 | [1] |  | primary |  | 1SF/>1SF | 120 | 240 | Clinical | ART  Mod.1 | 235 | 24 | 1 yr |
|  |  |  |  |  |  |  | 120 | 240 |  |  | 224 | 52 | 2 yrs |
|  |  |  |  |  |  |  | 120 | 240 |  |  | 218 | 58 | 3 yrs |
| Chen(b) | 2001 | [2] | A silver-amalgam made in China | primary | molar | 1SF | 104 |  | Clinical | (See  Part 2) | 52 | 9 | 1 yr |
|  |  |  |  |  |  |  |  |  |  |  | 21 | 7 | 2 yrs |
| She | 2003 | [3] |  | primary |  | 1SF/>1SF | 81 | 151 | Clinical | ART  Mod.2 | 130 | 13 | 1 yr |
|  |  |  |  |  |  |  | 81 | 151 |  |  | 108 | 15 | 2 yrs |
| Wang | 2004 | [4] | Silver amalgam by Shanghai Medical Instruments Co Ltd. | primary | molar | 1SF/>1SF | 100 | 153 | Clinical | (See  Part 2) | 153 | 33 | 1 yr |
|  |  |  |  |  |  |  |  |  |  |  | 142 | 47 | 2 yrs |
| Li(b) | 2005 | [5] | Silver amalgam by Shanghai Medical Instruments Co Ltd. | permanent |  | Class I |  |  | Clinical | (See  Part 2) | 52 | 16 | 2 yrs |
|  |  |  |  |  |  | Class II |  |  |  |  | 78 | 32 | 2 yrs |
| Ling | 2003 | [6] | Silver amalgam by China Iron & Steel Research Institute Group | primary | molar |  | 106 | 106 | Clinical | ART  Mod.3 | 106 | 22 | 1 yr |
|  |  |  |  |  |  |  | 106 | 106 |  |  | 106 | 33 | 2 yrs |
| Qiu | 2007 | [7] |  | primary |  | 1SF/>1SF | 64 | 190 | Clinical | ART  Mod.4 | 168 | 18 | 1 yr |
|  |  |  |  |  |  |  | 64 | 190 |  |  | 142 | 16 | 2 yrs |
| Li | 2002 | [8] |  | primary |  | 1SF/>1SF | 137 | 151 | Clinical | ART  Mod.5 | 121 | 13 | 1 yr |
|  |  |  |  |  |  |  | 137 | 151 |  |  | 105 | 15 | 2 yrs |
| Miranda | 2005 | [9] |  | primary | molar | 1SF |  | 36 | Clin/X-Ray | ARTC | 32 | 0 | 1 yr |
|  |  |  |  |  |  | >1SF |  | 44 |  |  | 42 | 4 | 1 yr |
| Taifour | 2002 | [10] | Avalloy | primary | molar | 1SF |  | 380 | Clinical | ARTC | 314 | 64 | 3 yrs |
|  |  |  |  |  |  | >1SF |  | 425 |  |  | 352 | 201 | 3 yrs |
| Yu | 2004 | [11] | GK-amalgam | primary | molar | Class I |  | 23 | Clinical | ARTC | 17 | 0 | 1 yr |
|  |  |  |  |  |  |  |  | 18 |  |  | 7 | 2 | 1 yr |
|  |  |  |  |  |  |  |  | 23 |  |  | 17 | 0 | 2 yrs |
|  |  |  |  |  |  |  |  | 18 |  |  | 7 | 2 | 2 yrs |
| Honkala | 2003 | [12] | Megalloy | primary | molar | Class I | 18 |  | Clinical | ARTC | 25 | 2 | 22 m |
|  |  |  |  |  |  | Class II | 18 |  |  |  | 10 | 0 | 22 m |
| Yip* | 2002 | [13] | GK-amalgam | permanent | molar | Class I |  | 29 | Clin/Indirect | USPHS | 22 | 0 | 1 yr |
|  |  |  |  |  |  |  |  | 29 | Clinical |  | 22 | 0 | 1 yr |
| Rahimtoola | 2002 | [14] | Tytin | permanent |  | (1SF) |  |  | Clinical | ARTC | 62 | 3 | 2 yrs |
| Frencken | 2006 | [15] | Avalloy | permanent |  | 1SF | 254 | 403 | Clinical | ARTC | 403 | 33 | 1.3 yrs |
|  |  |  |  |  |  |  | 205 | 403 |  |  | 323 | 34 | 2.3 yrs |
|  |  |  |  |  |  |  | 175 | 323 |  |  | 267 | 9 | 3.3 yrs |
|  |  |  |  |  |  |  | 145 | 267 |  |  | 218 | 27 | 4.3 yrs |
|  |  |  |  |  |  |  | 78 | 218 |  |  | 113 | 5 | 5.3 yrs |
|  |  |  |  |  |  |  | 75 | 113 |  |  | 108 | 11 | 6.3 yrs |
|  |  |  |  |  |  | >1SF | 13 | 33 |  |  | 33 | 7 | 1.3 yrs |
|  |  |  |  |  |  |  | 10 | 33 |  |  | 23 | 10 | 2.3 yrs |
|  |  |  |  |  |  |  | 5 | 23 |  |  | 9 | 3 | 3.3 yrs |
|  |  |  |  |  |  |  | 4 | 9 |  |  | 9 | 2 | 4.3 yrs |
| Yip | 2002 | [16] | GK-amalgam | primary | molar | Class I |  |  | Clinical | ARTC | 32 | 0 | 1 yr |
|  |  |  |  |  |  |  |  |  |  |  | 32 | 0 | 1 yr |
| Gao* | 2003 | [17] | GK-amalgam | permanent | molar | Class I |  | 29 | Clinical | USPHS | 6 | 0 | 30 mo |
| Estupiñán-Day | 2006 | [18] |  | permanent | molar |  |  | 888 | Clinical | USPHS | 755 | 43 | 1 yr |
|  |  |  |  |  |  |  |  | 677 |  |  | 617 | 6 | 1 yr |
|  |  |  |  |  |  |  |  | 232 |  |  | 211 | 12 | 1 yr |
|  |  |  |  |  |  |  |  | 888 |  |  | 624 | 70 | 2 yrs |
|  |  |  |  |  |  |  |  | 677 |  |  | 559 | 8 | 2 yrs |
|  |  |  |  |  |  |  |  | 232 |  |  | 211 | 15 | 2 yrs |
| Ye | 2006 | [19] | Silver amalgam | primary | molar | 1SF/>1SF | 68 | 174 | Clinical | ART /Mod.6 | 169 | 26 | 1 yr |
| Ling | 2003 | [20] | Silver amalgam by China Iron & Steel Research Institute Group | primary | molar | Class I |  |  | Clinical | (See  Part 2) | 106 | 22 | 1 yr |
|  |  |  |  |  |  |  |  |  |  |  | 106 | 33 | 2 yrs |

DS = Dataset; PG = Parallel group; SM = Splitmouth; m = Male; f = Female; ART = Atraumatic restorative treatment; AM = Amalgam; HEX = Hand excavation; SF = Tooth surface; X = Mean value; N = Number of evaluated teeth; n = Number of teeth with failed restorations; BSL = Baseline; yr(s) = Year(s); mo = Months; Clin = Clinical examination; Indirect = Silicon impression/plaster assessment; ARTC = ART criteria; USPHS - United States Public Health Service criteria; Ref. = Reference number; X-Ray = Radiological assessment

Evaluation of tooth restorations: ART- and modified ART criteria

| Score | ART criteria (ARTC)^1^ | Modification 1 (ART Mod.1) | Modification 2  (ART Mod.2) | Modification 3  (ART Mod.3) | Modification 4  (ART Mod.4) | Modification 5  (ART Mod.5) | Modification 6  (ART Mod.6) |
| --- | --- | --- | --- | --- | --- | --- | --- |
| 0 | Present, good. | Same as ARTC | Same as ARTC | Same as ARTC | Same as ARTC | Same as ARTC | Same as ARTC |
| 1 | Present, slight marginal defect for whatever reason, at any one place which is less than 0.5 mm in depth  No repair is needed. |  |  |  | Present, slight marginal defect for whatever reason, at any one place which is less than 1.0 mm in depth  No repair is needed. |  |  |
| 2 | Present, marginal defect for whatever reason, at any one place which is deeper than 0.5 mm but less than 1.0 mm in depth. Repair is needed. |  | Slight abrasion of the restoration margin (<0.5 mm) |  | Slight impairment of occlusal surface (<1 mm). No repair is needed. | Slight abrasion of the restoration margin (<0.5 mm) | Slight wear <0.5 mm |
| 3 | Present, gross defect of more than 1.0 mm in depth. Repair is needed. |  | Same as ART |  | Restoration and tooth intact but with slight marginal discoloration | Marginal damage on restoration. Repair is needed. | Marginal damage on restoration. Repair is needed. |
| 4 | Not present, restoration has (almost) completely disappeared. Treatment is needed. |  | Abrasion of the restoration margin. Repair is needed. | Present. Tooth fractured. | Present, wear and tear gradually over larger parts of the restoration, which is deeper than 1.0 mm. Repair is needed. | Marginal wear on restoration. Repair is needed. | Large wear on restoration. Repair is needed. |
| 5 | Not present, other restorative treatment has been performed. |  | Partly or completely lost | Partly or completely lost | Impairment of occlusal surface (>1 mm). Repair is needed. | Partly or completely lost | Partly or completely lost |
| 6 | Not present, tooth has been extracted. |  | Not present, other restorative treatment has been performed. | Not present, other restorative treatment has been performed. | Partly or completely lost | Not present, other restorative treatment has been performed. | Not present, other restorative treatment has been performed. |
| 7 | Present, wear and tear gradually over larger parts of the restoration but is less than 0.5 mm at the deepest point. No repair is needed. | Lost contact | Not present, tooth has been extracted. | Not present, tooth has been extracted. | Secondary caries | Not present, tooth has been extracted. | Tooth loss |
| 8 | Present, wear and tear gradually over larger parts of the restoration, which is deeper than 0.5 mm. Repair is needed. |  | Lost contact |  | Not present, other restorative treatment has been performed. | Lost contact | Lost to follow up |
| 9 | Unable to diagnose. |  |  |  | Not present, tooth has been extracted. |  |  |
| S | Score: 0, 1, 7 | 0, 1, 2 | 0, 1, 2 | 0, 1 | 0-3 | 0, 1, 2 | 0, 1, 2 |
| F | Failure: 2, 3, 4, 8 | 3-7 | 3-7 | 2-7 | 4-9 | 3-7 | 3-7 |

S = Restoration success; F = Restoration failure.

^1^As per: Frencken JE, Holmgren CJ. Atraumatic restorative treatment for dental caries. STI Book b.v. Nijmegen 1999, p. 58. [8]

references

[1] Chen B-X, Kang J, Guo N, Zhang S-L. A clinical study of atraumatic restorative tretament (ART) in children with dental caries (Article in Chinese). Acta Acad Med Jiangxi 2006; 46: 97-99.

[2] Chen X, Wei X-L. Clinical effects of glass ionomer cement FX in restoring deciduous molar teeth caries (Article in Chinese). Chin J Conserv Dent 2001; 11: 326-7.

[3] She X, Li X, Wan H, et al. A 2-year clinical trial of atraumatic restorative treatment in primary teeth (Article in Chinese). J Pract Stomatol 2003; 19: 30-33.

[4] Wang Q, Ding C-S. Evaluation of the effects of atraumatic restorative treatment in primary caries (Article in Chinese). J Taizhou Polytech Inst 2004; 4: 63.

[5] Li H-M, Dou Z-H. Clinical observation of using different material in the elderly decaying tooth ART technique (Article in Chinese). Pract Clin Med 2005; 6: 105-107.

[6] Ling L, Wang X. Evaluation of the effects of atraumatic restorative treatment and cooperation degree in primary caries (Article in Chinese). J Stomatol 2003; 23: 290-1.

[7] Qiuh N-L. Two-year clinical trial of atraumatic restorative treatment in primary teeth (Article in Chinese). Stomatol 2007; 27: 413-15.

[8] Li X, Hu D, Wan H et al. A two-year clinical trial of atraumatic restorative treatment in primary teeth (Article in Chinese). West Chin J Stomatol 2002; 4: 125-7.

[9] de Miranda LMS. Estudo clínico randomizado e controlado comparando o tratamento restaurador atraumático ao convencional com amálgama em molares decíduos: avaliação após 6 e 12 meses / Randomized and controlled clinical study comparing atraumatic restorative with amalgam conventional treatments in primary molars: 6 and 12 month evaluation.Thesis 2005; Rio de Janeiro: 220 p.

[10] Taifour D, Frencken JE, Beiruti N, van 't Hof MA, Truin GJ. Effectiveness of glass-ionomer (ART) and amalgam restorations in the deciduous dentition: results after 3 years. Caries Res 2002; 36: 437-44.

[11] Yu C, Gao XJ, Deng DM, Yip HK, Smales RJ. Survival of glass ionomer restorations placed in primary molars using atraumatic restorative treatment (ART) and conventional cavity preparations: 2-year results. Int Dent J 2004; 54: 42-6.

[12] Honkala E, Behbehani J, Ibricevic H, Kerosuo E, Al-Jame G. The atraumatic restorative treatment (ART) approach to restoring primary teeth in a standard dental clinic. Int J Paediat Dent 2003; 13: 172-9.

[13] Yip KH, Smales RJ, Gao W, Peng D. The effects of two cavity preparation methods on the longevity of glass ionomer cement restorations: an evaluation after 12 months. J Am Dent Assoc 2002; 133: 744-51.

[14] Rahimtoola S, van Amerongen E. Comparison of two tooth-saving preparation techniques for one-surface cavities. ASDC J Dent Child 2002; 69: 16-26.

[15] Frencken JE, Taifour D, van 't Hof MA. Survival of ART and amalgam restorations in permanent teeth of children after 6.3 years. J Dent Res 2006; 85: 622-6.

[16] Yip HK, Smales RJ, Yu C, Gao XJ, Deng DM. Comparison of atraumatic restorative treatment and conventional cavity preparations for glass-ionomer restorations in primary molars: one-year results. Quintessence Int 2002; 33: 17-21.

[17] Gao W, Peng D, Smales RJ, Yip KH. Comparison of atraumatic restorative treatment and conventional restorative procedures in a hospital clinic: evaluation after 30 months. Quintessence Int 2003; 34: 31-7.

[18] Estupiñán-Day S, Milner T, Tellez M. Oral health of low income children: procedures for atraumatic restorative treatment (PRAT) - Final report. PAHO 2006; Project number 091024: ATN/JF-7025-RG.

[19] Ye X, Liu T. The effect of ART in the restoration of deciduous molars (Article in Chinese). J Dent Prev Treat 2006; 14: 39-40.

[20] Ling L, Wang X. Observation on effects of atraumatic restorative treatment on adjacent occlusal caries at primary molars (Article in Chinese). Guangdong Odontopath Prev 2003; 11: 40-41.
